# Proteolysis-Targeting Chimeras (PROTACs) in Cancer Therapy: Present and Future

**DOI:** 10.3390/molecules27248828

**Published:** 2022-12-12

**Authors:** Rui Li, Miao Liu, Zhenya Yang, Jiao Li, Yuxin Gao, Ruirong Tan

**Affiliations:** 1 Sichuan Cancer Hospital and Institute, School of Medicine, University of Electronic Science and Technology of China, Chengdu 610041, China; 2 Department of Pathology, Brigham and Women’s Hospital, Harvard Medical School, Boston, MA 02115, USA; 3 Xiangya School of Pharmaceutical Sciences, Central South University, Changsha 410083, China; 4 Translational Chinese Medicine Key Laboratory of Sichuan Province, Sichuan Institute for Translational Chinese Medicine, Sichuan Academy of Chinese Medicine Sciences, Chengdu 610041, China

**Keywords:** PROTACs, cancer therapy, targeted protein degradation, protein degradation

## Abstract

The PROteolysis TArgeting Chimeras (PROTACs) is an innovative technique for the selective degradation of target proteins via the ubiquitin–proteasome system. Compared with traditional protein inhibitor drugs, PROTACs exhibit advantages in the efficacy and selectivity of and in overcoming drug resistance in cancer therapy, providing new insights into the discovery of anti-cancer drugs. In the last two decades, many PROTAC molecules have been developed to induce the degradation of cancer-related targets, and they have been subjected to clinical trials. Here, we comprehensively review the historical milestones and latest updates in PROTAC technology. We focus on the structures and mechanisms of PROTACs and their application in targeting tumor-related targets. We have listed several representative PROTACs based on CRBN, VHL, MDM2, or cIAP1 E3 ligases, and PROTACs that are undergoing anti-cancer clinical trials. In addition, the limitations of the current research, as well as the future research directions are described to improve the PROTAC design and development for cancer therapy.

## 1. Introduction

Despite the continuous development of new treatment methods, malignant tumors still seriously endanger human health [1]. Compared with traditional chemoradiotherapy and surgical treatments, individually targeted approaches and immunotherapy have been large breakthroughs for the treatment of malignant tumors over the past 20 years. Currently, they are highly active areas of research. At present, the molecularly targeted drugs that have been used in clinical treatments are mainly small molecule inhibitors and monoclonal antibodies, which bind to the active sites of the target proteins as competitive antagonist ligands to hinder the target protein’s ability to bind to their downstream targets. However, drug resistance may occur due to changes in the binding sites caused by gene mutations or alterations of the conformation of the target protein. At present, though traditional targets (such as kinases and G protein-coupled receptors) are still the main research focus, the landscape of the drug target field is changing, slowly shifting from traditional drug targets to more challenging “undruggable” targets. These targets typically include proteins that have no enzymatic function which account for about 80% of the total human proteins.

PROTACs, PROteolysis-TArgeting Chimeras, are heterobifunctional small molecule compounds, which consists of a ligand for the target protein, a linker, and a ligand to recruit E3 ligase. It can induce the degradation of the target proteins, including the “undruggable” targets. It is a new strategy for the research and development of novel small molecule drugs, and it can address the dilemma of the known problems of using targeted drugs [2]. At present, several PROTAC drugs have undergone or are undergoing clinical trials, including ARV-110 [3] which targets the androgen receptor in prostate cancer, ARV-471 [4], targeting the estrogen receptor in breast cancer, and FHD-609 which targets BRD9 in Synovial sarcoma. In this paper, we introduce the structure and mechanism of the PROTACs, summarize the research progress and clinical application of PROTACs with different E3 ligases in cancer treatments, and describe the limitations of the current research and perspectives for the future development of PROTACs.

## 2. Structure Character and Mechanism of PROTACs

The main pathway for protein degradation in the cells is ubiquitin–proteasome system-mediated degradation. E3 ubiquitin ligase recognizes and ubiquitinates the target proteins, and the ubiquitinated target proteins enter the 26S proteasome for degradation [5]. On the basis of the biological rationale of ubiquitination-mediated degradation, proteolytic targeting chimera (PROTAC) has been developed. Many targets that cannot be regulated by small molecules or antibodies can be affected by PROTAC technology. Unlike the traditional role of small molecule drugs which aim to block or inhibit the function of proteins, the novelty and usefulness of PROTAC is its ability to send any target proteins into the proteasome for degradation [6].

**Figure 1:**
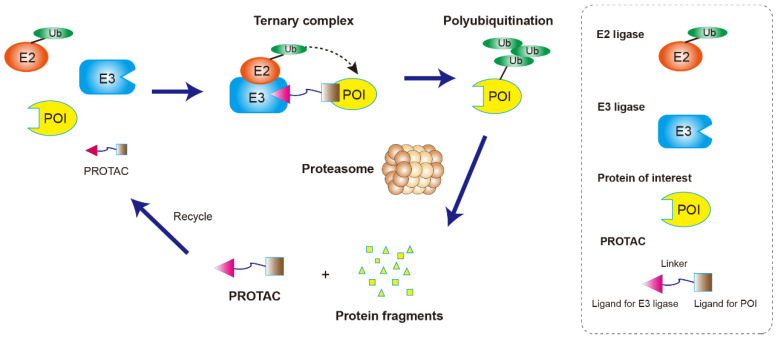
Illustration of the PROTAC mechanism. PROTACs are composed of a ligand that binds to a protein of interest (POI) which is tethered to another ligand that binds to the ubiquitin ligase enzyme through a short chemical linker. A POI-PROTAC-E3 ligase ternary complex is formed which leads to tagging of the protein of interest with ubiquitin groups. The proteasome then recognizes the polyubiquitination signal and mediates the degradation of the target protein. The PROTAC molecule can be recycled for another degradation cycle.

As a heterobifunctional molecule, PROTAC is composed of three parts (Figure 1): a target protein ligand, an E3 ligase ligand, and a linker that connect the two ligands to form a “three-body” polymer, the target protein ligand-linker-E3 ligand. It uses the ubiquitin–proteasome pathway to specifically degrade the target protein by bringing the target protein closer to the E3 ubiquitin ligase in the cell [3]. As shown in Figure 1, PROTAC-induced POI degradation includes four major steps: Firstly, PROTAC binds to the POI and E3 ligase to form a ternary complex (POI-PROTAC-E3 ligase). Secondly, E3 ligase recruits E2 ligase and mediates the transfer of ubiquitin (Ub) from the E2 to lysine residues on the POI. Thirdly, the ubiquitination occurs several times to form multi-Ub chains on the surface of the POI. Finally, the ternary complex dissociates, and the polyubiquitinated POI is degraded by the 26S proteasome. The dissociated PROTAC can then participate in a new round of degradation [7].

**Figure 2:**
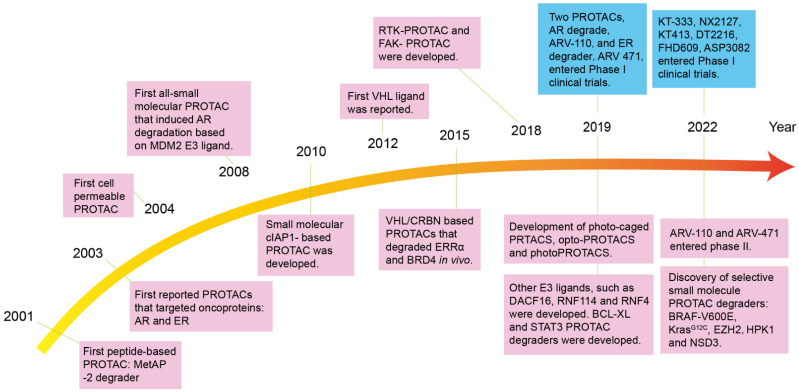
Timeline of PROTACs technology [9,12,15,25]. The concept of PROTAC was first proposed in 2001 when Sakamoto et al. [8]. developed the first peptide-based PROTAC. Since then, PROTAC technology has flourished, from peptide-based to small molecule-based PROTACs which adopt different types of E3 ligases to the later improved small-molecule PROTACs such as photoPROTAC [93]. In 2019, the results of Phase I clinical trials of ARV-110 [3], an AR inhibitor, and ARV-471 [4], an ER inhibitor, confirmed the feasibility and efficacy of PROTACs in degrading pathogenic proteins to treat human diseases. An increasing number of PROTACs are now designed to target different disease-causing proteins, and they have begun to enter the clinic, which hopefully will provide meaningful benefits to cancer patients [95].

In 2001, Sakamoto et al. [8] first proposed the concept of PROTACs. They successfully designed and synthesized the first PROTAC bifunctional molecule for the degradation of methionine aminopeptidase 2 (MetAP-2). At first, the PROTAC molecule was mainly composed of peptides. The PROTAC-1 molecule targeting MetAP-2 was composed of a module that binds to MetAP-2, the angiogenesis inhibitor ovalicin, and a module that binds to the E3 ubiquitin ligase complex SCF (Skp1-Cullin-Fbox), a phosphopeptide (IPP) from the IKBα protein that is specifically recognized by β-TRCP. It was then demonstrated that PROTAC-1 could recruit MetAP-2 and SCFβ-TRCP [9]. Since this initial result, the PROTAC technique has been advanced, and different PROTACs have been designed for many proteins, such as the BET protein family [8], EGFR [10], and aperiodic cyclin dependent kinases (CDKs) [11]. Many of these proteins are associated with the growth and metastasis of malignant tumors, and they are existing or potential therapeutic targets. Although CRISPR, siRNA, small molecule inhibitors, monoclonal antibodies, and other methods and strategies have been developed to regulate the expression or function of tumor-related proteins, PROTAC has many advantages including diversified targets, a fast action speed, and a high specificity for the selective degradation of pathogenic variant proteins without affecting the functions of normal proteins [12,13]. PROTAC has shown great potential in depleting pathogenesis-related proteins and treating malignant tumors (Figure 2) [14,15]

## 3. The Application of PROTACs in Anti-Cancer Drug Discovery and Development

### 3.1. Peptide-Based PROTAC

The first generation of PROTACs employed the peptide motif as the ligand of E3 ubiquitin ligase. Peptide-based PROTACs have high biocompatibility and low toxicity in vivo [16]. Previous studies have confirmed that the target proteins that could be degraded by peptide-based PROTACs include the estrogen receptor (ER) [17], androgen receptor (AR) [18], methionyl aminopeptidase 2(MetAP2) [8], and protein kinases (AKT, PI3K) [19]. As mentioned above, in 2001, the ubiquitin ligase SCFβ-TRCP (SKP1-CUL1-Fbox) was used as a part of PROTAC, and its effectiveness was verified in cells. By replacing the ovalicin of PROTAC-1 targeting METAP-2 with estradiol, which can bind to the estrogen receptor (ER), or dihydroxytestosterone (DHT), which can bind to the androgen receptor (AR), they attempted to induce the targeted degradation of ER, a breast cancer-associated protein, and AR, a prostate cancer-associated protein, respectively. The ability of these PROTACs to degrade ER and AR was experimentally confirmed, and this result suggested that PROTACs could be used to degrade specific tumor-associated target proteins to treat malignant tumors. However, PROTACs with E3 ligands in the form of polypeptides have large molecular weights, and they cannot freely cross the cell membrane. Sakamoto et al. [8] used microinjection to introduce the PROTACs into the cells, a technique that is not useful in clinical settings. The unsuitable characteristics of the peptide-based PROTACs, including their high molecular weight, poor cell penetration, and potential low stability [20], have restricted their application.

The use of cell-penetrating peptides (CPPs) to mediate the entry of biological macromolecules into the cells is a research hotspot in the field of biomedicine [21]. Schneekloth et al. [22,23] first reported in 2004 PROTACs, named PROTA-4 and PROTAC-5, which can independently penetrate the cell membranes. The E3 ubiquitin ligase was converted from the SCF system to the von Hippel-Lindau elongin B and C-Cul2 complex. At the same time, the ligand linking pVHL (the von-Hippel-Lindau protein) in VBC-Cul2 was changed into a heptapeptide (ALAPYIP), and a D-type arginine polymer was linked to the pVHL to enhance its membrane permeability, reduce the rate of degradation, and enhance its effect. The ligand for the VHL E3 ligase used in this study was a peptide derived from HIF-1α (Hypoxia-inducible factor 1α), a VHL substrate. In subsequent studies, VHL has become one of the most commonly used E3 complexes in PROTAC applications [24].

Since then, peptidyl PROTAC has been continuously optimized to target cancer-related proteins [25]. For example, polyarginine has been used to improve the membrane’s permeability. In a study on the targeting of hepatitis B virus X protein by a peptidyl PROTAC containing polyarginine to treat hepatocellular carcinoma showed that the X protein could be significantly degraded in the cancer cells [26]. Further advancement was made with the identification of a shorter peptide of HIF-1α [27], and the researchers designed and synthesized a phosphorylated polypeptide PROTAC molecule [28]. In 2013, Crews and colleagues [19] demonstrated in a mouse model that PhosphoPROTACs, molecules based on a cell transmembrane peptide, can inhibit tumor cell growth by targeting the degradation of Phosphatidylinositol 3-kinase (PI3K). This was the first report on the therapeutic effect of PROTACs in vivo. These findings strongly suggested that PROTAC may be used in the treatment of malignant tumors. However, the polypeptide structure of the PROTAC molecules still has some shortcomings, such as a large molecular weight and unstable peptide bonds, and its application as antitumor drugs still needs to be explored [9].

### 3.2. Small-Molecule-Based PROTACs

In order to overcome the weakness of peptide-based PROTACs, small molecule-based PROTACs have been rapidly developed. Compared with peptide-based PROTAC, small molecule-based PROTACs are more easily absorbed by the cells in the body, and they are more promising to be developed into drugs [29]. Their research has also moved from in vitro cell experiments to animal and animal disease model experiments. Some small molecule drugs have entered clinical trials. More than 600 E3 ligases have been identified in humans, and the common ones include MDM2 (mouse double minute 2 homologue), cIAP1 (cellular inhibitor of apoptosis protein 1) [30], CRBN (cereblon) [31], and VHL (von Hippel-Lindau) [32]. These ligases are usually multi-subunit E3 ligase complexes, whose structure is conducive to the ligands’ design and optimization.

#### 3.2.1. MDM2-Based PROTACs

p53 is an indispensable tumor suppressor gene in the human body, and its mutation is an important driving factor for the occurrence, development, treatment, resistance, and poor prognosis of a variety of malignant tumors [33]. The mouse double minute (MDM2) protein is the major negative cellular regulator of p53, and it is classified as an oncogene because it suppresses p53 activity. Nutlins are cis-imidazoline analogs with high affinity with MDM2 and can regulate p53 activity by inducing the competitive disruption of MDM2-p53 binding [34]. Therefore, nutlin is often used in cancer treatments [35]. Previous studies have found that MDM2, as a ligand of E3 ligase in PROTACs, can be used to degrade AR [22] and BRD4 [36]. In 2008, Schneekloth et al. [22]. creatively combined Nutlin-3a with a Selective androgen receptor modular (SARM) to target AR. By this action, they synthesized the first complete small molecule form of PROTAC. It was confirmed that it could effectively degrade AR in the Hela cells in a proteosome-dependent manner at a concentration of 10mM. Thus, the feasibility of PROTAC as a patent drug was generally recognized by the industry. In addition, Hines et al. [24] used idasanutlin as a MDM2 ligand and JQ1 (a thienotriazolodiazepine) as a bromodomain and extraterminal 4 (BRD4/BET) inhibitor to form PROTAC-A1874, which could degrade the target protein with an efficiency of 98%, which is in the nanomolar concentration range. It is worth noting that A1874 can degrade BRD4 and stabilize p53 at the same time, thus showing a better intracellular effect than the corresponding VHL-based PROTAC can, which acts on the same target protein. This was the first report of the synergistic inhibition of proliferation byan E3 ligase ligand and a targeting warhead.

#### 3.2.2. VHL-Based PROTACs

VHL mainly functions as a tumor suppressor gene, encoding two isotypes of the protein pVHL. It is a member of a protein complex as described above, and it can promote the degradation of HIF-1α and other protein targets required for cell growth and angiogenesis [37,38]. It is also the first E3 ligase that was widely used in small molecule-mediated degradation [39]. The early VHL E3 ligases were peptide-based PROTACs, which were constructed from the HIF-1α peptide sequence with their membrane permeability being improved by using a polyarginine linker, but they had disadvantages such as a low efficiency and a lack of stability. Therefore, the research work of VHL E3 ligase was mainly focused on finding suitable and efficient small-molecule substitutes for the HIF-1α polypeptide fragments [40].

In 2012, Crews group [41,42] synthesized a series of novel small-molecule inhibitors against VHL using rational design. On this basis, Bondeson et al. [43] synthesized the first VHL E3-recruited small-molecule PROTACs by conjugating the thiazolidinedione-based selective estrogen-associated receptor (ERRα) ligand with the VHL ligand to act on ERRα and RIPK2. They subsequently showed that the PROTACs could efficiently degrade ERRα and RIPK2 both in vitro and in mice. Thus far, a variety of VHL E3-based PROTACs have been used for the targeted degradation of different disease-related proteins, such as BRD4 [44], BCL-ABL [45], ALK [46], RTK [47], BRD7/9 [48], FAK [49], smad3 [50], ER, and AR [51], as well as others.

BRD4 (bromodomain protein 4) is a transcriptional regulator and a member of the BET family, which has become an emerging therapeutic target for malignant tumors due to its important role in cell cycle regulation and differentiation. Zengerle et al. [44] first combined JQ1 with the VHL E3 ligand to form the PROTAC molecule MZ1, which was used for the degradation of the BET family of proteins, and it verified its inhibitory effect on proliferation in several types of leukemia cells. Compared with BRD2 and BRD3, MZ1 is highly selective for BRD4. Through analyzing the complex crystal structure of MZ1 with the human VHL and Brd4 bromide domains, Gadd et al. [52] designed and synthesized AT1, which possessed a higher efficiency than MZ1 did, further verifying the usefulness and effectiveness of PROTAC. ARV-771 [53] is another VHL-based small-molecule PROTAC designed and synthesized by Raina K and his colleagues as a Pan-BET inhibitor. Compared with the conventional BET inhibitors, ARV-771 has higher efficiency in colorectal cancer cell lines, and it can simultaneously inhibit the expression of AR protein and the transmission of the AR signal. Further studies showed that an ARV-771 treatment could reduce the leukemia burden to a greater extent and further improve the survival of NSG mice transplanted with the HEL92.1.7 cell line when they are compared with the BET inhibitor OXT015 [54].

The BCR-ABL fusion gene produces the structural protein BCR-ABL, which is the main cause of chronic myeloid leukemia (CML) [55]. Clinically, tyrosine kinase inhibitors targeting BCR-ABL can be used to treat CML, but they are prone to drug resistance and off-target effects [56]. Using an E3 ligase VHL PROTAC, along with a series of small molecules that they developed, the Crews team [51] was able to specifically degrade the BRC-ABL fusion proteins. Lai et al. [45] reported a PROTAC targeting BCR-ABL, and they confirmed that BCR-ABL could be degraded in in vitro cell experiments, and as well as this, they found that the types of PROTAC target ligands and E3 ubiquitin ligase recruiting elements had a great influence on the degradation efficiency of BCR-ABL. The targeted PROTAC SIAIS178, which is based on dasatinib, a second-generation BCR-ABL inhibitor, can not only efficiently degrade the BCR-ABL protein, but it can also successfully induce the significant regression of xenograft tumors in vivo [57]. This offers the possibility of improving the drug resistance of the small-molecule inhibitors of CML in the future.

#### 3.2.3. CRBN-Based PROTACs

In 2010, the researchers studying the mechanism of action of the immunomodulator thalidomide discovered that its main target is cereblon (CRBN), the E3 component of the Culin-RING ubiquitin ligase (CRL) complex [58]. Since then, thalidomide and its derivatives lenalidomide and pomalidomide have attracted great interest as potent immune modulators, and they have been approved for the treatment of multiple myeloma. Mechanistically, the immunomodulatory drugs (IMiDs) target the E3 ubiquitin ligase CUL4-RBX1-DDB1-CRBN, also known as CRL4 CRBN. The binding of the IMiDs to the complex allows for the recruitment of IKAROS family transcription factors (IKZF1 and IKZF3) and ubiquitination on these endogenous substrates for therapeutic effects.

The Crews team [59] created the small-molecule PROTAC ARV-825 by linking the targeted drug candidate OTX-015 with pomalidomide through a PEG chain to degrade the BRD4 protein, and its effective concentration was in the nanomolar range. Compared with the BRD4 inhibitors JQ1 and OTX-015 alone, ARV-825 was more effective in inhibiting tumor cell proliferation and inducing apoptosis. Winter et al. [60] created dBET1 by linking the BET inhibitor JQ1 with the thalidomide derivatives, which could induce the degradation of BRD4 protein in a human leukemia cell line AML, while the inactive dBET1 did not degrade the protein. dBET1 also leads to degradation of other BET family members (BRD42, BRD3) with poor specificity. In addition, although ARV-825 has a similar structure to dBET1, its efficiency in inducing BRD4 degradation is 10 times higher than that of dBET1, which may be mainly due to the difference in the chain-linkage between the two molecules. As well as this, QCA570 [61] and BETd-260 [62], composed of the CRBN E3 ligand and newly screened BET small molecule inhibitors, have also been used for cancer treatment.

Bruton tyrosine kinase (BTK) is a key regulator in the B cell antigen receptor (BCR) signal transduction pathway, which is related to the differentiation and maturation of the B cells, and it is a potential therapeutic target for B cell malignancies [63]. Ibrutinib, a covalent inhibitor of BTK, can treat hematological malignancies by inhibiting BTK phosphorylation. However, the mutation of C481S at the ibrutinib binding site often leads to clinical drug resistance [64]. Ibrutinib can also inhibit ITK and EGFR and induce cellular toxicity. PROTAC P13I synthesized by combining ibrutinib, a BTK inhibitor, with pomalidomide can degrade wild-type and mutant C481S BTK, simultaneously, without inhibiting ITK or EGFR, and thus, it can eliminate the side effects. Yu Rao’s team [65] synthesized PROTAC L18I, a small molecule targeting BTK and its mutants, based on ibrutinib and lenalidomide, another CRBN ligand. It was also verified that it could exert an anti-tumor effect by degrading BTK in a mouse xenograft tumor model constructed using HBL-1 cells carrying the C481S mutation.

The tyrosine kinase receptor, ALK, was first discovered in chromosomal translocations associated with anaplastic large cell lymphoma, and it is associated with the occurrence and progression of a variety of cancers, such as non-small cell lung cancer, neuroblastoma, renal cell carcinoma, as well as others [66]. Soda M et al. [67] found that the EML-ALK fusion gene was the driver gene of non-small cell lung cancer, and the clinical studies also confirmed that crizotinib, an ALK inhibitor, could treat non-small cell lung cancers which showed a positive expression of ALK [68]. Another group synthesized new PROTAC molecules degraders, and they verified their feasibility to degrade ALK in multiple tumor cell lines. Since then, many small-molecule PROTACs have been developed to fight cancer by degrading ALK, such as MS4077 and MS4078 [46], TD-004 [69], etc.

Cyclin-dependent kinases (CKDs) are conserved serine/threonine kinases in eukaryotes, which are involved in the regulation of the cell cycle by binding to cyclin isoforms [70]. Given the key role of CDKs in the cell cycle, gene transcription, and other biological processes, it has been an important target for cancer therapies. The selective CDK4/6 inhibitor Palbociclib was approved by the FDA for the treatment of ER-positive and HER2-negative advanced breast cancer patients [71]. Georg E Winter [60] first designed and synthesized the PROTAC molecule BSJ-03-123, which selectively degrades CDK6 instead of CDK4 through the CRBN E3 ubiquitin ligase. Moreover, CDK6 can be effectively degraded, and the proliferation of the tumor cells can be inhibited in CDK6-dependent and CKD4-independent acute myeloid leukemia cells. In order to investigate the important roles of the ligand binding affinity, adaptor, spatial orientation, E3 ligase ligand, and cell permeability in the PROTAC’s function, Su et al. [72] designed and synthesized a focused PROTAC library for CDK6.

The induced myeloid leukemia cell differentiation protein (MCL-1) gene is one of the key members of the BCL-2 family, and it is highly expressed in many cancers of the blood, such as lymphoma and leukemia. The MCL-1 protein plays a role in cancer by regulating cell apoptosis through protein–protein interaction (PPI). MCL1 was once categorized as a non-druggable protein. J.W. Papatzimas et al. [73] reported that a PROTAC based on E3 ligase CRBN could significantly reduce the amount of MCL1. Afterwards, the dual Mcl-1/Bcl-2 inhibitor Nap-1 was linked to pomalidomide to achieve the simultaneous knockdown of Mcl-1 and Bcl-2 [74]. PPI has been a difficult problem in the field of small-molecule ligands, and PROTAC technology provides a powerful opportunity to target PPIs.

With the development of PROTAC research, in addition to the above-mentioned PROTACs, some other PROTACs based on the E3 ligase CRBN have been reported, such as PROTACs targeting PI3K [75], AKT [76], HDAC6 [77], STAT3 [78], etc.

##### 3.2.4. cIAP1-Based PROTACs

cIAP1 and cIAP2 are members of the Inhibitor of Apoptosis Proteins (IAPs) family of proteins identified from Tumor Necrosis Factor Receptor (TRAF2)-related proteins, and they play an important role in the negative regulation of apoptosis [79]. In this family, cIAP1 is overexpressed in many tumor cells, and it is considered to be a promising target for cancer treatments. In addition, cIAP1 itself is an E3 ligase that promotes ubiquitination and subsequent proteasome degradation [80].

Itoh et al. [30] designed and synthesized a small-molecule PROTAC, targeting the degradation of intracellular retinoic acid binding protein 1/2 (CRABP-1/2) using cIAP1 inhibitor methyl bestatin as the E3 ligase ligand. However, the studies have shown that this small-molecule PROTAC has many limitations, such as the ability of these SNIPERs to induce auto-ubiquitination of the IAP, which can lead to the degradation of cIAP1 itself, in addition to the degradation of the target protein, and because bestatin itself is an aminopeptidase inhibitor, its off-target effects often bring about adverse reactions [81]. To overcome these defects, they synthesized a new series of SNIPERs by altering the attachment site of the MeBS, which could specifically degrade CRABP-2 without affecting cIAP1 [82]. Additionally, PROTACs based on SPNIPERs/IAPs are also anticipated to exert antitumor activity in those tumor cells that evade apoptosis by upregulating the IAPs.

## 4. PROTACs in Clinical Trials

Compared with the small-molecule inhibitors, PROTAC has obvious advantages such as targeting the “undruggable” targets, the ability to overcome drug resistance, a prolonged action time, activity in smaller doses, a good safety profile, and more specific selectivity [83]. In 2019, Arvinas Therapeutics used a heterobifunctionally targeted degrader for the first time in human trials, which entered clinical phase I. So far, more than a dozen PROTACs including BCL-xL, IRAK4, STAT3, BTK, BRD9, MDM2, and other targets have entered the clinical research and the development stage, and most of them are first-in-class targets [84] (Table 1).

The phase I clinical results of ARV-110 and ARV471 confirmed the efficacy, safety, and druggability of the PROTACs. ARV-110 is the earliest PROTAC protein-lowering agent that was developed in the world, which targets the androgen receptor (AR), and it is used for the treatment of metastatic castration-resistant prostate cancer (mCRPC). The phase I results (NCT03888612) showed that a dose of 420 mg was well tolerated in humans, and this demonstrated for the first time the PROTAC-mediated degradation of target proteins in humans. At present, it is in phase II clinical trials, and the disclosed phase II clinical results show that it can reduce the prostate specific antigen (PSA) level by ≥50% (PSA50) in 46% of the patients with the AR T878X/H875Y mutation. Among seven patients who met the RECIST (Response Evaluation Criteria in Solid Tumors) criteria and could be assessed, six patients experienced tumor reduction, and two patients showed a confirmed partial response (PR). Therefore, it has shown initial efficacy in treating tumors [3].

The PROTAC drug, ARV-471, which targets ER (estrogen) receptor degradation, is designated for use in patients with locally advanced or metastatic ER-positive HER2 (human epidermal growth factor receptor 2)-negative breast cancer. The phase 1 data from ARV-471 show that high levels of ER degradation (89%) were observed in the enrolled patients at all of the dose levels from 30 mg to 700 mg, and these were well tolerated. ARV-471 is currently in phase II clinical trials as a single agent for advanced metastatic breast cancer, and a phase Ib study has been initiated to evaluate the combination of ARV-471 with the CDK4/CDK6 agent palbociclib in advanced metastatic breast cancer [4].

Based on the highly positive results of PROTAC in AR and ER clinical studies, in recent years, the PROTAC targeting of different target proteins has also begun to enter clinical studies. DT2216 [85], which was constructed by coupling ABT263 (an inhibitor of BCL-2 and BCL-XLdual) with the VHL ligand, is a highly efficient small-molecule PROTAC for the degradation of BCL-XL, and it has entered phase I clinical studies for T cell lymphoma and small-cell lung cancer. FHD-609 (NCT04965753), which acts as a BRD9 target, is also being studied in Synovial sarcoma cases. KT-333, which targets STAT3, was granted an orphan drug status by the FDA in the United States for the treatment of peripheral T cell lymphoma (PTCL) [86]. PROTAC breaks through the development problem of traditionally “undruggable” targets, and it provides a new definition of small-molecule drugs. As a revolutionary technology in the field of biomedicine, new PROTACs will continue to enter clinical research [25].

## 5. Challenges in PROTACs Study

To date, PROTACs have been successfully applied in the degradation of many protein targets. However, there are still some limitations to PROTACs that need to be overcome through future research. Due to their special structures, the PROTACs usually have a high molecular weight and a poor performance in pharmacokinetics. Although many PROTACs have been reported to exhibit activities in vivo, and they are clinically studied now due to their special structures, the PROTACs usually have a high molecular weight and a poor performance in pharmacokinetics and druggability studies [87]. The toxicity and side effects of PROTACs are also a large concern in terms of their application in the clinic. On one hand, unlike the traditional inhibitors which only inhibit the activity, but do not affect the expression of the target proteins, the PROTACs induce the degradation of the target protein, by which drug resistance may be prevented but a higher irreversible toxicity could be generated [88]. On the other hand, the off-target effect of the PROTACs may induce severe consequences for affecting the physiological activities of the normal cells or organs. Therefore, the close monitoring and tracking of the toxicological effects are needed as for the clinical studies and in the late stages of the drug development of PROTACs [89].

## 6. Perspectives

The strategy of using PROTACs in treating malignant tumors is very promising. PROTACs utilize a highly effective cellular process (degradation) on target proteins and have shown better results than chemical inhibitors, using very low dosages without the side effect of inducing drug resistance [90]. They could also be recycled in the body, and they have the ability to target “undruggable” targets. Therefore, they have great potential and highly optimistic prospects for a variety of cancer treatments [91]. Future studies on several aspects related to PROTACs would greatly improve and promote the application of PROTACs in treating diseases: (1) They could improve the cells’ permeability. PROTACs have poor cell permeability, especially for the blood–brain barrier. The strategies based on small-molecule prodrugs with low molecular weight, such as the CLIPTACs technique [92], could significantly improve the permeability and solubility of the drugs in the cells. In this case, there would be no need to optimize the linker. (2) They could improve the targeting ability. The addition of manipulating the elements of the PROTACs to control them to exert effects only at specific time points and locations would help to elevate their targeting ability and reduce toxicity. Light-inducible PROTACs have shown potential to improve their targeting ability [93]. Pan et al. [94] have developed a light-inducible PROTAC, which they called pc-PROTAC, which could control the effect of the PROTAC and improve its targeting ability. (3) They could improve their flexibility. The targeting ability of PROTACs mainly depends on the ligands (peptides) which specifically bind to the target proteins. The identification of the suitable ligands for novel target proteins is challenging, and this has restricted the development of PROTACs. PROTACs with fusion tags have been developed to improve their flexibility. For example, Crews et al. [51] have induced the HaloTag (HT) for PROTACs and developed a HaloPROTAC system, which uses the combination of E3 ligand and chlorinated paraffin to degrade the HT fusion protein. It is believed that these novel techniques may produce great improvement in the application of PROTACs and the discovery of novel anticancer drugs in the near future.

## Figures and Tables

**Table 1:** PROTACs in clinical trials.

Degrader	Target	E3 ligase	Indications	NCT Numbers (If Applicable)	Phase	Company	ROA	Start Year
**CC-94676**	AR	CRBN	Prostate cancer	NCT04428788	Phase Ⅰ	Bristol Myers Squibb	Oral	2020.6
**HP518**	AR	Undisclosed	Metastatic castration-resistant prostate cancer	NCT05252364	Phase Ⅰ	Hinova Pharmaceuticals	Oral	2021.12
**ARV-766**	AR	Undisclosed	Prostate cancer	NCT05067140	Phase Ⅰ	Arvinas	Oral	2021.9
**AC176**	AR	Undisclosed	Prostate cancer	NCT05241613	Phase Ⅰ	Accutar Biotech	Oral	2022.1
**ARV-110**	AR	CRBN	Prostate cancer	NCT03888612(U.S.) NCT05177042(Canada)	Phase Ⅱ	Arvinas	Oral	2019.3(U.S.) 2021.11(Canada)
**DT2216**	BCL-XL	VHL	Liquid and solid tumors	NCT04886622	Phase Ⅰ	Dialectic Therapeutics	I.V.	2021.4
**FHD-609**	BRD9	Undisclosed	Synovial sarcoma	NCT04965753	Phase Ⅰ	Foghorn Therapeutics	I.V.	2021.6
**CFT8634**	BRD9	CRBN	Synovial sarcoma	NCT05355753	Phase Ⅰ/Ⅱ	C4 Therapeutics	Oral	2022.4
**NX-5948**	BTK	CRBN	B cell malignancies and autoimmune disease	NCT05131022	Phase Ⅰ	Nurix Therapeutics	Oral	2021.11
**NX-2127**	BTK	CRBN	B cell malignancies	NCT04830137	Phase Ⅰ	Nurix Therapeutics	Oral	2021.3
**BGB-16673**	BTK	Undisclosed	B cell malignancy and lymphoma	NCT05294731(China) NCT05006716(U.S.)	Phase Ⅰ	BeiGene	Oral	2022.2(China) 2021.8(U.S.)
**HSK29116**	BTK	Undisclosed	Relapsed/refractory B cell malignancies	NCT04861779	Phase Ⅰ	Haisco	Oral	2021.8
**CFT8919**	EGFR-L858R	CRBN	Non-small-cell-lung cancer		IND-e	C4 Therapeutics	Oral	
**ARV-471**	ER	CRBN	Breast cancer	NCT04072952(U.S.) NCT05463952(Japan) NCT05501769(U.S.)	Phase Ⅱ	Arvinas/Pfizer	Oral	2019.8(U.S.) 2022.7(Japan) 2022.8(U.S.)
**AC682**	ER	CRBN	Breast cancer	NCT05080842(U.S.) NCT05489679(China)	Phase Ⅰ	Accutar Biotech	Oral	2021.9(U.S.) 2022.7(China)
**KT-413**	IRAK4	CRBN	Diffuse large B cell lymphoma (MYD88-mutant)	NCT05233033	Phase Ⅰ	Kymera	I.V.	2022.1
**ASP3082**	KRAS G12D	Undisclosed	Solid tumor	NCT05382559	Phase Ⅰ	Astellas Pharma	Intravenous infusion	2022
**KT-333**	STAT3	Undisclosed	Liquid and solid tumors	NCT05225584	Phase Ⅰ	Kymera	Undisclosed	2021.12
**CG001419**	TRK	CRBN	Cancer and other indications		IND-e	Cullgen	Oral	

AR, androgen receptor; BCL-XL, B Cell Lymphoma-extra-large; BRD9, bromodomain-containing protein 9; BTK, Bruton’s tyrosine kinase; EGFR-L858R, the L858R mutant form of the epidermal growth factor receptor; ER, estrogen receptor; IRAK4, interleukin-1 receptor-associated kinase 4; KRAS G12D, Kirsten Rat Sarcoma Viral Proto-Oncogene G12D mutation; STAT3, signal transducer and activator of transcription 3; TRK, tropomyosin receptor kinase; CRBN, cereblon; VHL, von Hippel–Lindau; IND-e, in IND-enabling preclinical studies; ROA, route of administration; I.V., intravenous.

## Data Availability

Not applicable.
